# Determinants of IGF-II influencing stability, receptor binding and activation

**DOI:** 10.1038/s41598-022-08467-8

**Published:** 2022-03-18

**Authors:** Andrew Blyth, Michael Ortiz, Allanah Merriman, Carlie Delaine, Briony Forbes

**Affiliations:** Department of Medical Biochemistry, Flinders Health and Medical Research Institute, Flinders University, Bedford Park, SA 5042 Australia

**Keywords:** Biophysical chemistry, Peptide hormones, Biochemistry, Peptides

## Abstract

Insulin like growth factor II (IGF-II) is involved in metabolic and mitogenic signalling in mammalian cells and plays important roles in normal fetal development and postnatal growth. It is structurally similar to insulin and binds not only with high affinity to the type 1 insulin-like growth factor receptor (IGF-1R) but also to the insulin receptor isoform A (IR-A). As IGF-II expression is commonly upregulated in cancer and its signalling promotes cancer cell survival, an antagonist that blocks IGF-II action without perturbing insulin signalling would be invaluable. The high degree of structural homology between the IR and IGF-1R makes selectively targeting either receptor in the treatment of IGF-II-dependent cancers very challenging. However, there are sequence differences between insulin and IGF-II that convey receptor selectivity and influence binding affinity and signalling outcome. Insulin residue YB16 is a key residue involved in maintaining insulin stability, dimer formation and IR binding. Mutation of this residue to glutamine (as found in IGF-II) results in reduced binding affinity. In this study we sought to determine if the equivalent residue Q18 in IGF-II plays a similar role. We show through site-directed mutagenesis of Q18 that this residue contributes to IGF-II structural integrity, selectivity of IGF-1R/IR binding, but surprisingly does not influence IR-A signalling activation. These findings provide insights into a unique IGF-II residue that can influence receptor binding specificity whilst having little influence on signalling outcome.

## Introduction

**Figure 1:**
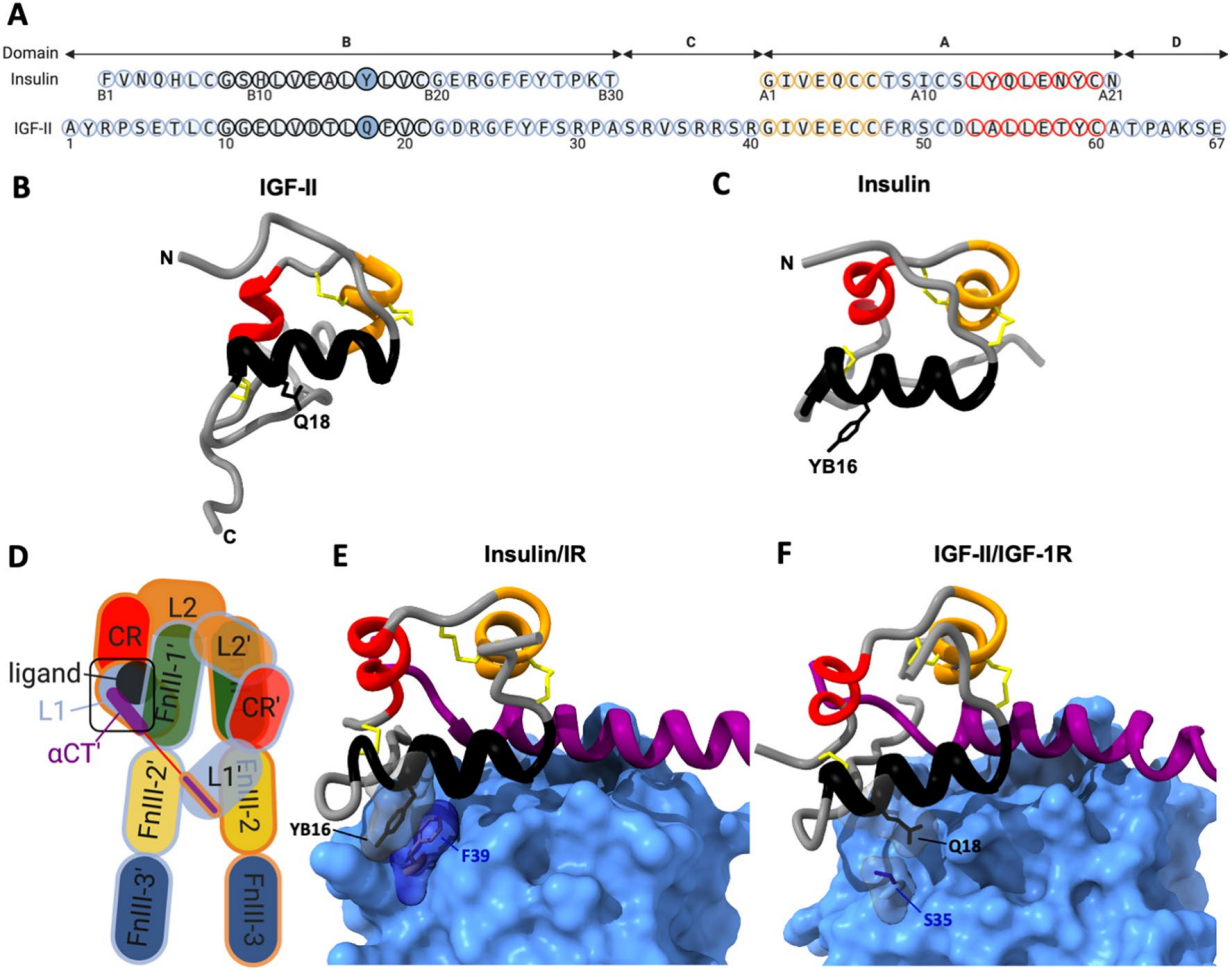
Sequence and structural comparison of IGF-II and insulin. (**A**) Sequence alignment of IGF-II and insulin. Domains are indicated above. Each peptide has three alpha helices; B-chain helix 1 (black circles), A-chain helix 2 (orange circles) and A-chain helix 3 (red circles). Residue numbers are indicated below each sequence. Insulin YB16 and IGF-II Q18 residues are in solid filled circles. (**B**) IGF-II and (**C**) insulin ribbon structures (PDB: 1IGL and 1MSO respectively) show the three disulphide bonds and helices coloured as in (**A**). Side chains of YB16 in insulin and Q18 in IGF-II are shown. (**D**) Schematic diagram representing the IR and IGF-1R ligand bound extracellular domain structures. Individual αβ monomer outlines are coloured either blue or orange. Extracellular domains include the first and second leucine-rich repeat domains (L1 and L2), cysteine-rich domain (CR), first, second and third fibronectin type-III domains (FnIII-1, -2, and -3), insert domain (ID), α-chain C-terminal region (αCT). Transmembrane and intracellular domains, including the tyrosine kinase domain, are not shown. (**E**) Insulin and (**F**) IGF-II bound to site 1 of their cognate receptors, as highlighted with the black box in (**D**) (from PDB: 6HN5 and 6VWI, respectively). L1 domains are surface filled in cornflower blue, α-chain C-terminal regions (αCT) are coloured purple. The side chains of insulin residue YB16 and F39 of the IR L1 domain and the equivalent residues Q18 in IGF-II and S35 of the IGF-1R L1 domain are shown with transparent surface fill.

Insulin like growth factor II (IGF-II)^1^ plays important roles in regulation of embryonic growth and development as well as adult growth^1^. IGF-II is most abundant during fetal development and is well established as a key regulator of growth and development at this time^2,3^. IGF-II and insulin are highly similar in structure (Fig. 1A,B) and function, with both controlling metabolic and mitogenic responses in mammalian cells. The IGF/insulin system comprises three structurally similar tyrosine kinase receptors: the type 1 Insulin-like growth factor receptor (IGF-1R) and the two splice variants of the insulin receptor (IR-A and IR-B isoforms). Upon ligand binding to these receptors two main signalling pathways are activated: the PI3K/Akt metabolic pathway and the ERK/MAP kinase mitogenic signalling pathway. IGF-II binds with high affinity to both the IGF-1R and the IR-A leading to activation of mitogenic cell growth and survival responses^4^. Insulin acts via both IR isoforms, with IR-B signalling being responsible for metabolic regulation and IR-A signalling resulting in mitogenic responses. As IGF-II is overexpressed in several forms of cancer including brain, thyroid and ovarian cancers^1,5^, and IGF-II signalling promotes cancer cell growth and survival, this signalling pathway is an important target for development of cancer therapies. With the ultimate goal of developing specific inhibitors or antagonists of IGF-II action in cancer, a detailed understanding of the molecular mechanisms underlying IGF-II action in normal and abnormal cell growth is required.

Whereas the molecular mechanisms of insulin action at the insulin receptor have been extensively studied, far less is known about the structural determinants and biophysical properties of IGF-II that dictate its function. Both IGF-II and insulin comprise homologous B- and A-domains with 47% sequence similarity. Mature insulin is a two-chained 51 amino acid peptide held together by the one intra chain (A6-A11) and two inter chain (A7-B7, A20-B19) disulfide bonds, whereas IGF-II is a single-chain 67 amino acid peptide, with B- and A-domains linked by a connecting C-domain (Fig. 1A). Folding of insulin, and presumably IGF-II, into the native conformation involves initial formation of the B19-A20 disulfide bond (IGF-II C21–C60) and a folding nucleus, with the remaining two disulfide bonds (IGF-II C9-C47 and C46-C51) forming rapidly thereafter^6^. The overall tertiary structures are highly similar with each comprising three α-helices (Fig. 1B,C)^7–9^. Through specific determinants in the B- and A-chains insulin forms dimers and is stored as zinc coordinated hexamers in pancreatic islets^10^. In its monomeric form insulin has a strong propensity to aggregate^11^. In vitro IGF-II also has a tendency to aggregate^7^ although the structural determinants responsible have not been determined.

A series of studies have implicated the insulin residue YB16 positioned within the insulin B-chain α-helix as a key residue in ligand structural stability and dimerization. It plays a role in maintenance of the B-chain helix structure, with mutation to proline perturbing disulfide pairing and impairing secretion in mammalian cells^12^. Furthermore, deletion of this residue has been linked to neonatal diabetes, presumably due to a similar perturbation of the B-chain helical structure^13^. Not only is the residue involved in maintenance of structural integrity but it also plays a role in insulin dimerization. Mutation to histidine or alanine results in a monomeric insulin^14,15^. In IGF-II the equivalent residue 18 is a non-aromatic glutamine and in this study we explore whether it has similar functions in stability and aggregation.

Our previous studies have identified sequence differences between IGF-II and insulin that account for their receptor binding specificities^17^. Determinants in the IGF-II B-, A- and C-domains contribute to high affinity binding to the IGF-1R and also account for the eight–tenfold lower affinity for the IR-A compared to insulin^16,17^. Site-directed mutagenesis studies of the IGF-II and insulin ligands have defined two receptor binding sites located on opposite surfaces of the ligand. The so-called “site 1” has been characterized as the primary binding site and involves a high affinity interaction with the receptor, whereas site 2 residues have been characterized as contributing to a low affinity interaction^18–20^. The site 1 surfaces play similar roles in binding to both the IGF-1R and the IR^21,22^.

The IGF-1R and IR have identical domain structures (Fig. 1D) comprising 2α and 2β subunits. Ligand binding mediates major structural rearrangement of the receptors from an open ‘Λ-shape’ to the activated ‘J-shape’^4,21–23^. How specific interactions between particular ligand residues and the receptor result in activation of the downstream signalling pathways is only partially understood. Structural studies of the ligand-bound receptors have clearly defined the ‘primary’ site 1 interaction with the L1 domain from one monomer and the αCT’ domain from the opposite monomer (Fig. 1D,E,F)^4^. There is also a low affinity interaction with receptor FnIII-1’ domain from the opposite monomer involving one of the previously defined site 2 residues (E12 IGF-II or HB10 insulin)^4^. The remaining ligand site 2 residues do not contact the receptor in the ‘J-shaped’ fully activated structure and have been proposed to be involved in a transient interaction prior to the final active signalling conformation^21–27^.

Insulin residue YB16 is involved in site 1 contact with the IR (Fig. 1E). Mutation to either alanine or histidine results in a two–threefold decrease in IR binding affinity^14,15^. A change to glutamine (as found in IGF-II) leads to a significant (tenfold) decrease in binding affinity for the IR-A^28^. This highlights a point of difference between the interaction of insulin and IGF-II with the IR. The YB16 of insulin contacts residue F39 of the L1 domain of the IR-A through a high affinity π–π interaction, as revealed by recent cryo-electron microscopy (cryoEM) structures (Fig. 1E)^21^. The change in insulin to a non-aromatic glutamine is unable to substitute for the tyrosine to generate such a high affinity interaction. Interestingly the glutamine substitution also results in a significantly reduced metabolic potency, suggesting this interaction influences IR activation^28^, although mutation to alanine has only minor impact on IR metabolic activity^14^.

As YB16 plays such an important role in the biophysical and functional properties of insulin we sought to understand whether the equivalent residue Q18 has a similar impact on the structure and function of IGF-II. Unexpectedly, mutation of Q18 to tyrosine had a major effect on analogue production due to aggregation problems and also the mutation affected IGF-IIs thermal stability. We uncover a unique role of IGF-II residue 18 as a determinant of receptor binding specificity, but surprisingly changes at Q18 do not influence signalling outcomes.

## Results

### Production and characterization of IGF-II analogues

**Figure 2:**
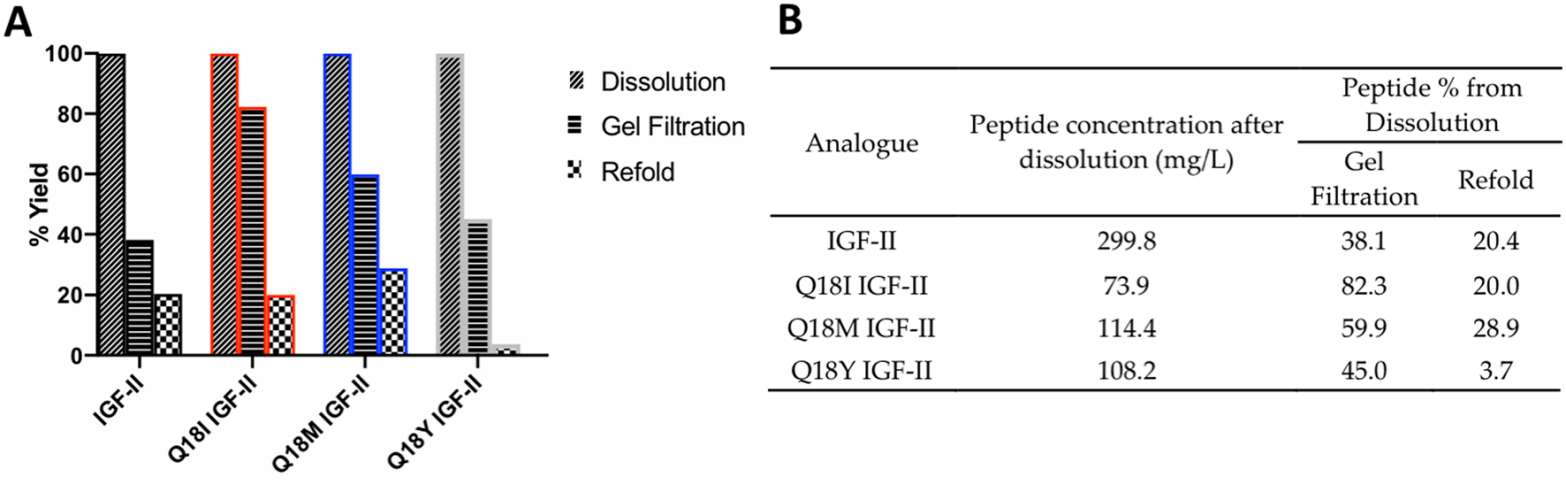
(**A**) Concentration at gel filtration and refold steps in the purification of Q18 IGF-II analogue fusion proteins. Yield is expressed as a percentage of the concentration after dissolution (100%) (data from Fig. 2A presented in **B**). (**B**) Table of protein concentration at dissolution (mg/L) and then yield at subsequent steps expressed as a percentage of each of the analogue concentrations at dissolution.

To investigate the roles of IGF-II residue Q18 in the biophysical properties, receptor binding and receptor signalling three IGF-II analogues were produced; Q18I IGF-II, Q18M IGF-II and Q18Y IGF-II. Each IGF-II analogue was expressed in *E. coli* as a fusion protein, including 11 N-terminal amino acids of porcine growth hormone and a cleavage recognition site, refolded and the mature peptide purified^29^ at sufficient quantities for biophysical and functional assays. Mass spectrometry confirmed correct masses for each analogue (Table. S1). Expression efficiencies of fusion peptide analogues in *E. coli* were similar (Supplementary Fig. S1) and equivalent to native IGF-II (not shown). Figure 2 summarizes the protein yield at each step of analogue production. Notably, the concentration of all IGF-II fusion peptide analogues at the dissolution stage was markedly lower than for the IGF-II fusion peptide (Fig. 2A,B). Thereafter the yields post gel filtration were 38% (IGF-II) and above for all peptides. At the completion of refold, yields were similar between IGF-II (20.4%) Q18I (20.0%) and Q18M IGF-II (28.9%) (refer to Supplementary Fig. S2-S5 for analytical HPLC profiles of each analogue during dissolution, gel filtration and refold). However, Q18Y IGF-II fusion peptide yield after refold compared to at dissolution was considerably lower (3.7%) than for IGF-II (20.4%). This loss of Q18Y IGF-II fusion protein is likely due to precipitation, which was visually evident during refold. This observation suggests that the Q18Y mutation is poorly tolerated, and only a small proportion of protein is capable of folding into the energetically favorable native-like conformation.

### Circular dichroism studies

**Figure 3:**
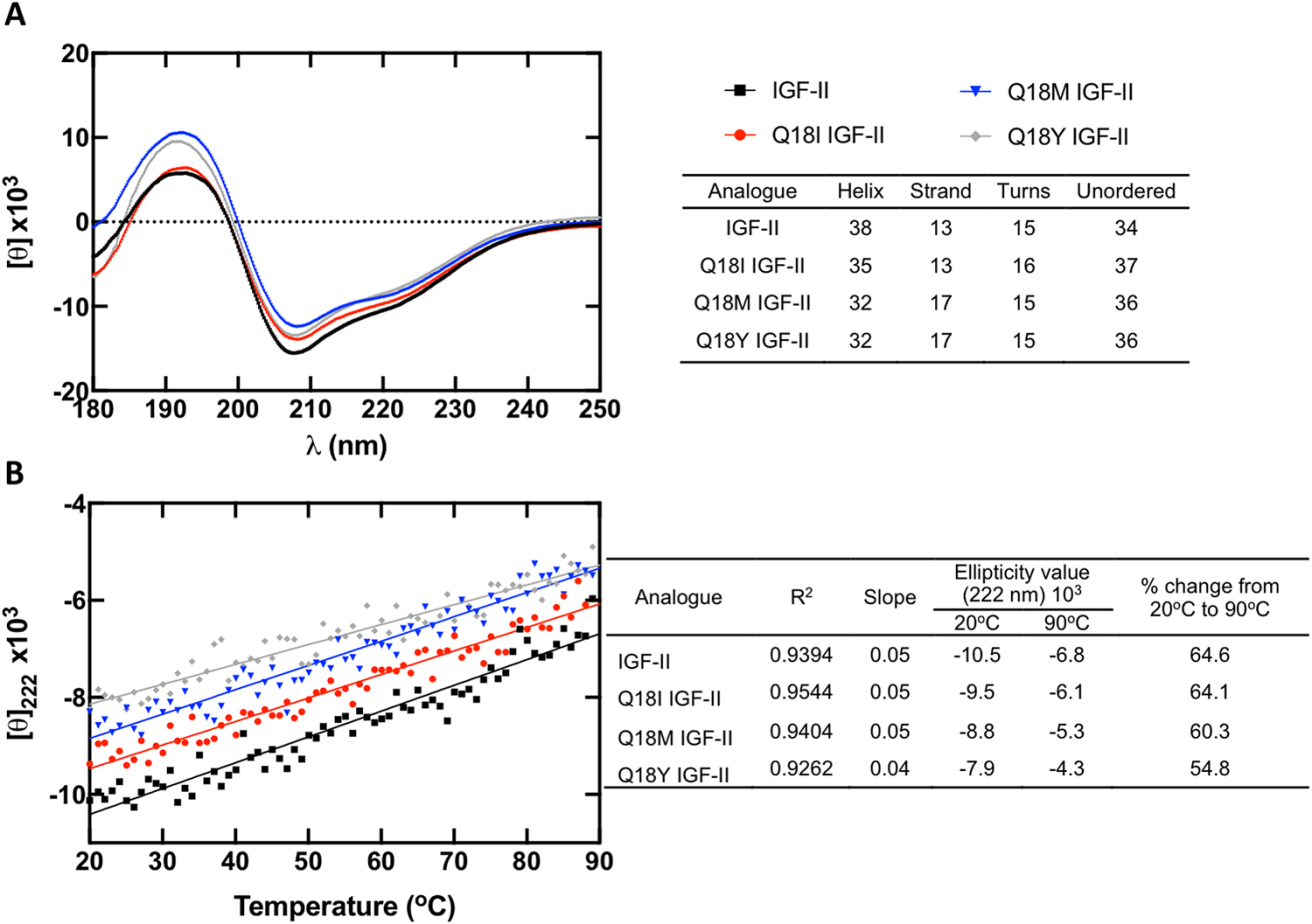
(**A**) CD spectra of IGF-II (black), Q18I IGF-II (red), Q18M IGF-II (blue) and Q18Y IGF-II (grey). Percent helical content shown in the table was calculated using the CDSSTR algorithm for deconvolution against the reference protein database set SMP180. (**B**) Temperature denaturation assays monitored by ellipticity at 222 nm.

Circular dichroism (CD) spectra were recorded between 180 and 250 nm (Fig. 3A). Q18I, Q18M and Q18Y IGF-II spectra all exhibited attenuated ellipticity at 222 nm, compared to IGF-II, indicating partial loss of α-helical content^30^. The α-helical content calculated based on the CD spectra obtained for IGF-II was 38%, consistent with previous reports^7^. The-helical content of Q18I IGF-II was calculated to be 35%, whereas Q18Y and Q18M IGF-II were found to have equal α-helical content of 32%.

### Temperature denaturation

Temperature denaturation assays were performed from 20–90 °C, for each analogue (Fig. 3B) and α-helical content was monitored by CD at the helix sensitive wavelength 222 nm. Consistent with the 222 nm values obtained for the CD spectra (Fig. 3A) IGF-II had the lowest ellipticity, followed by Q18I, Q18M and Q18Y IGF-II. As shown in Fig. 3B, the rate of change (slope) in ellipticity with increasing temperature is similar between Q18I, Q18M IGF-II and IGF-II (also expressed as % change from 20 to 90 °C in the accompanying table), whilst the rate of change in ellipticity for Q18Y IGF-II was the lowest at this temperature, indicating this analogue is the least thermodynamically stable.

### Immunocaptured receptor binding assays

**Figure 4:**
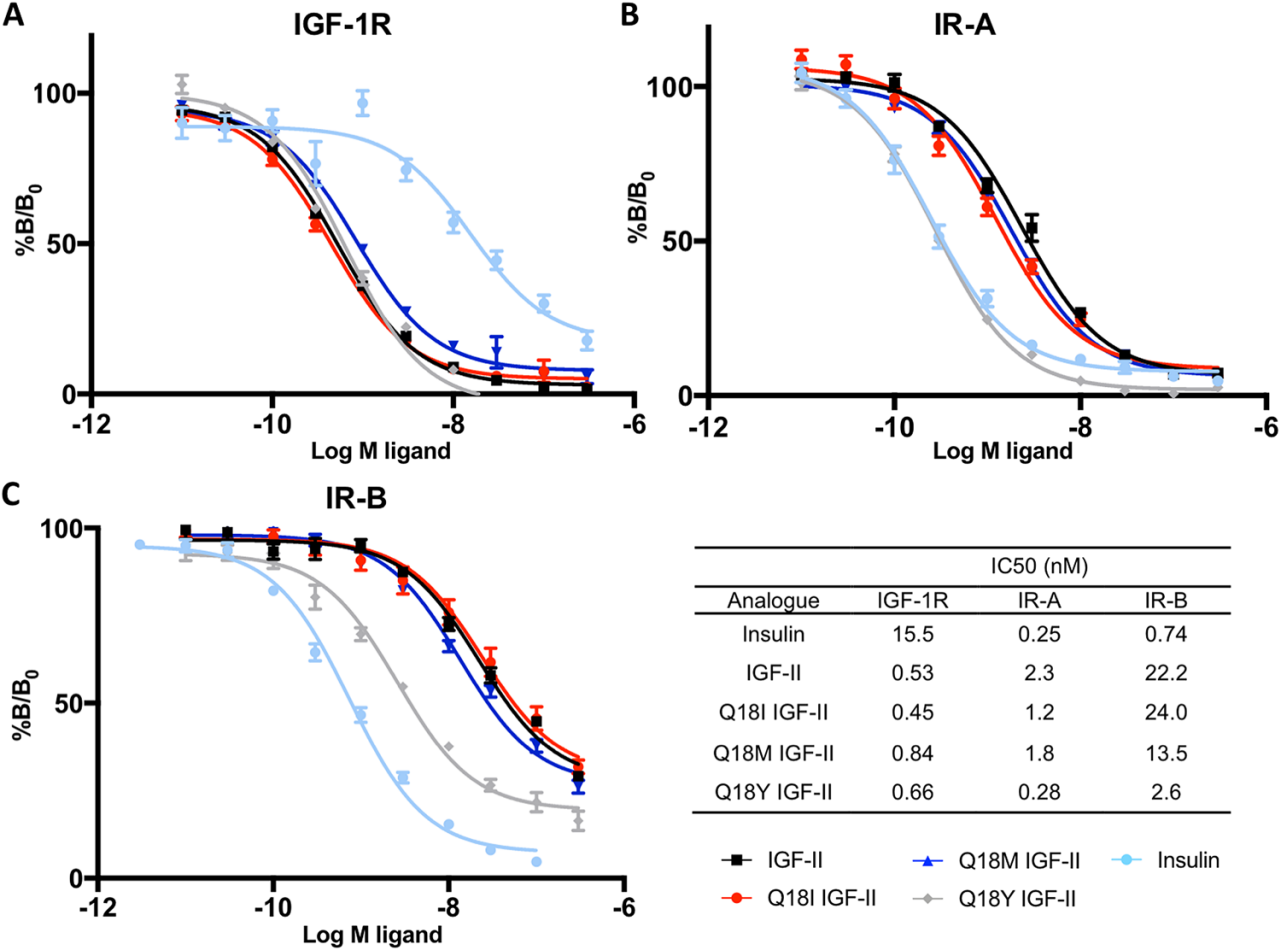
(**A**) Competition binding of insulin, IGF-II and IGF-II analogues with europium-labelled IGF-II for the IGF-1R (A) and IR-A (**B**), and europium-labelled insulin for the IR-B (**C**). Immunocaptured IGF-IR, IR-A or IR-B were incubated with europium-labelled IGF-II or insulin and increasing concentrations of the competitive ligands insulin, IGF-II, Q18I IGF-II, Q18M IGF-II or Q18Y IGF-II. Time-resolved fluorescence was measured with 340 nm excitation and 612 nm emission filters using a Multilabel Reader. Results are expressed as a percentage of binding in the absence of competing ligand (%B/B_0_). Data shown are the mean ± S.E.M. bars are shown where greater than the size of the symbols. n =  ≥ 3 independent experiments each with triplicate technical replicates. IC_50_ values for each ligand are shown in the table.

IGF-1R binding assays were performed using europium (Eu) labelled IGF-II and increasing concentrations of competing ligands (Fig. 4A). As expected, insulin had a low affinity for the IGF-1R with an IC_50_ value of 15.5 nM, which is similar to previous reports using Eu labelled IGF-I^31^. All three Q18 analogues bound the IGF-1R with similar affinities to IGF-II (IC_50_ 0.53 nM). Q18I IGF-II had the highest binding affinity for the IGF-1R, with an IC_50_ value of 0.45 nM, and Q18M and Q18Y IGF-II had similar IC_50_ values of 0.84 and 0.66 nM, respectively.

IR-A binding assays were also performed using Eu labelled IGF-II (Fig. 4B). As expected, insulin bound with a ninefold higher affinity to the IR-A than IGF-II, consistent with current literature^5,31,32^. Interestingly, there was little effect on binding with the Q18I and Q18M substitutions (Fig. 4B), whereas substitution with tyrosine resulted in an 8.2-fold increase in affinity compared to IGF-II, to an IC_50_ similar to insulin. A similar observation was made in IR-B binding assays performed with Eu labelled insulin and increasing concentrations of competing ligands (Fig. 4C). Insulin bound with a 30-fold higher affinity to the IR-B than IGF-II, consistent with current literature^31^. Again, there was minimal effect of the isoleucine and methionine substitutions on IR-B binding, whereas Q18Y had an 8.5-fold increased binding affinity compared to IGF-II. However, Q18Y IGF-II still did not bind IR-B as well as insulin (a 3.6-fold difference in affinities). Overall, these binding assays highlight the importance of the tyrosine residue in high affinity binding to the insulin receptor.

### Activation of Akt and ERK signalling

**Figure 5:**
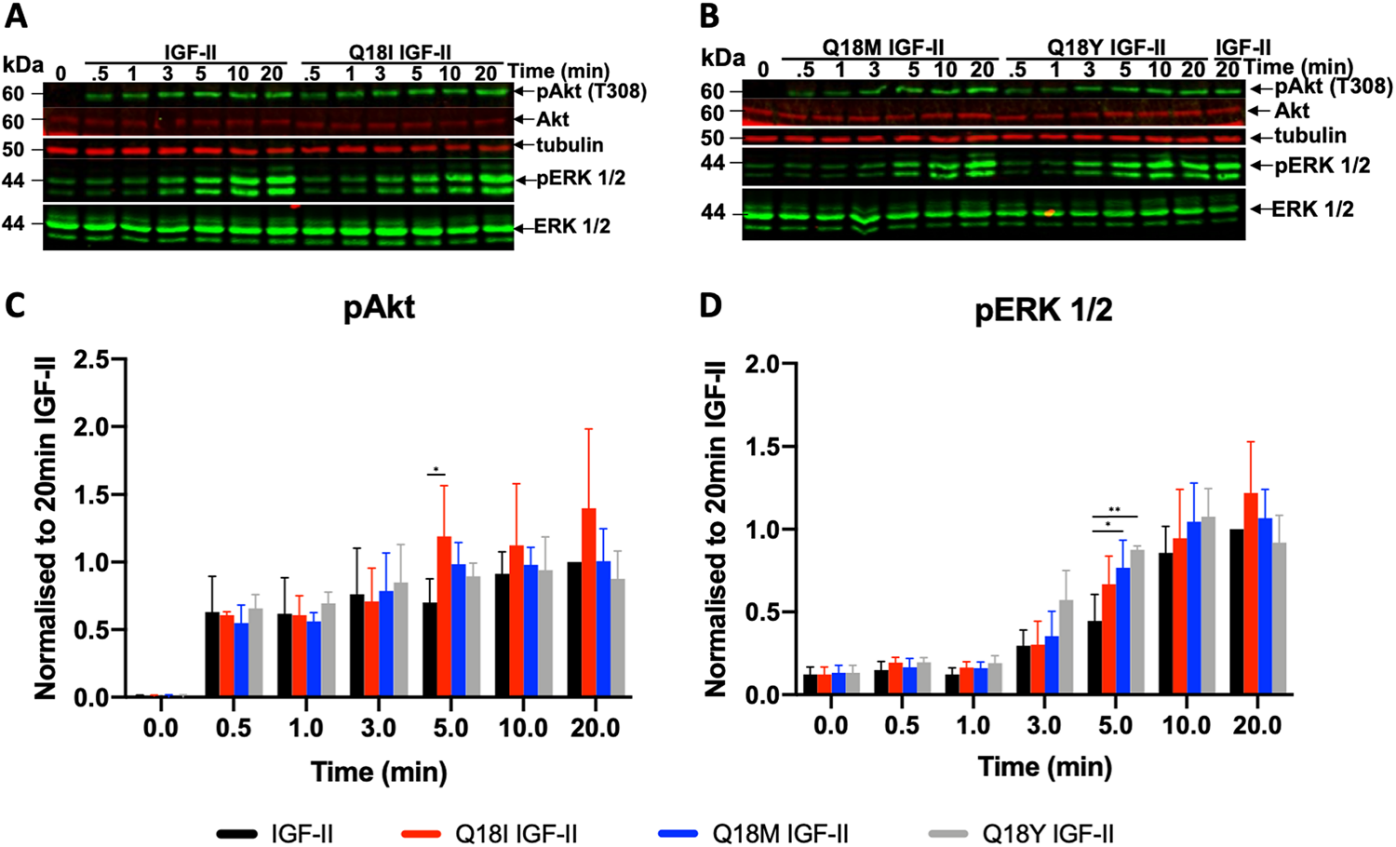
Induction of Akt and ERK phosphorylation upon IR-A activation by IGF-II and Q18 IGF-II analogues. L6 IR-A cells were treated with IGF-II, Q18I IGF-II, Q18M IGF-II, or Q18Y IGF-II at 10 nM for 0, 0.5, 1, 3, 5, 10, 20 min. Whole cell lysates were prepared and subjected to SDS-PAGE followed by immunoblotting for phosphorylated Akt (pAkt), total Akt, phosphorylated ERK 1/2 (pERK 1/2) and total ERK 1/2 (**A**,**B**). Each blot included lanes from cells untreated (basal = 0 min) and treated with 10 nM IGF-II for 20 min. Densitometric quantitation of n =  ≥ 3 independent experiments are shown as a bar graph of the mean ± S.E.M. Relative pAkt (**C**) and pERK (**D**) are expressed as a fraction of the level detected when cells were stimulated with 10 nM IGF-II for 20 min. There is no change in total Akt and ERK 1/2 at these time points. In each case, pAkt and pERK 1/2 was first normalized against the loading control (β-tubulin). Refer to Supplementary Fig. S6 for uncropped Western Blot images. A two-way ANOVA with Dunnett’s multiple comparison was performed. The only significantly different pAkt responses in (**C**) were IGF-II versus Q18I IGF-II at t = 5 min (*p value 0.01 to 0.05). The only significantly different pERK 1/2 responses in (**D**) were at t = 5 min for IGF-II versus Q18M IGF-II (*p value 0.01 to 0.05 and IGF-II versus Q18Y IGF-II **p ≤ 0.01).

Potency of activation of metabolic and mitogenic signalling was also measured through immunoblotting phosphorylated Akt (T308) and ERK respectively (Fig. 5). L6 rat skeletal myoblasts overexpressing the human IR-A (L6 IR-A) were treated with 10 nM of either Q18 IGF-II analogues or IGF-II and a time course was performed (0,0.5, 1, 3, 5, 10 and 20 min). Each IGF-II analogue was equipotent to IGF-II at stimulating Akt phosphorylation (Fig. 5A,C), except for Q18I IGF-II at t = 5 when there was a small but significant difference to IGF-II (p = 0.01 to 0.05). Each IGF-II analogue was similarly found to be equipotent to IGF-II in stimulating ERK phosphorylation (Fig. 5B,D), except for t = 5 min Q18M IGF-II (p = 0.01 to 0.05) and Q18Y IGF-II (p ≤ 0.01) when there was a small but significant difference to IGF-II. It was surprising that Q18Y IGF-II was essentially equipotent to IGF-II despite its 8.2-fold greater IR-A binding affinity.

### DNA synthesis assay

**Figure 6:**
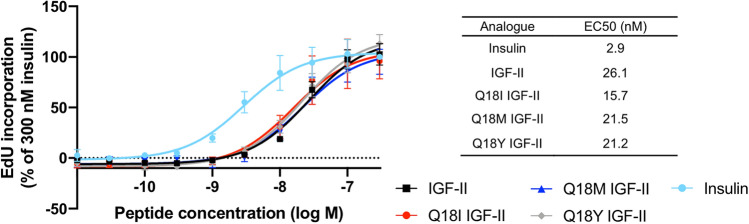
DNA synthesis in response to increasing concentrations of insulin, IGF-II and IGF-II analogues. Serum starved L6 rat skeletal myoblasts overexpressing the IR-A were treated with 0.01–300 nM insulin, IGF-II, Q18I IGF-II, Q18M IGF-II, or Q18Y IGF-II for 18 h. Cells were incubated for 4 h with 10 µM 5-Ethynyl-2’-deoxyuridine (EdU). 2 µM FAM-Azide 488, 100 mM Tris, pH 7.5, 4 mM CuSO_4_, 100 mM sodium ascorbate was added to the cells for 30 min. Fluorescence was measured using 485 nm excitation and 535 nm emission filters using a Multilabel Reader. Data is shown as a percentage incorporation of 300 nM Insulin. Data shown are the mean ± S.E.M. n =  ≥ 3 independent experiments. A two-way ANOVA with Dunnett’s multiple comparison was performed. Insulin versus IGF-II (****p < 0.0001).

Mitogenic potency of each analogue was measured using a DNA synthesis assay performed using L6 IR-A cells. Insulin potently activated DNA synthesis, ninefold more potently than IGF-II (Fig. 6). All IGF-II analogues were equipotent to IGF-II in activating DNA synthesis. This result is consistent with the equal ability of the analogues to activate ERK1/2 (and suggests the small differences seen at 5 min (Fig. 5B,D) are not biologically significant), and also demonstrates the unexpected low potency of Q18Y IGF-II despite its IR-A binding affinity being equal to insulin.

## Discussion

There is extensive understanding of the biophysical and functional properties of insulin and the roles played by individual residues in receptor binding and activation, whereas there is relatively little known about the determinants of IGF-II aggregation, stability and function through the IGF-1R and IR-A. Here we sought to ascertain whether the residue Q18 plays similar roles as the equivalent YB16 residue of insulin, which is involved in maintaining structural stability and insulin dimerization as well as being key in receptor binding. Three analogues of IGF-II, Q18I IGF-II, Q18M IGF-II and Q18Y IGF-II were expressed as fusion peptides in *E. coli* in inclusion bodies. Interestingly the fusion peptides of all analogues were more difficult to solubilize than IGF-II fusion peptide and more peptide remained as insoluble aggregate at this step. Also, the refold step for the Q18Y IGF-II fusion peptide was significantly less efficient than for all other peptides due to marked precipitation observed under the refold conditions. Seemingly the presence of a hydrophobic residue, and in particular the aromatic ring structure of tyrosine, at this position in the B-chain promotes self-association. This is consistent with the role of the equivalent residue B16 of insulin in dimerization, as demonstrated by substitution with alanine or histidine, rendering insulin monomeric^14^. One would predict that introduction of glutamine into position B16 of insulin would reduce self-association, although this has not been reported.

A previously unrecognized role for Q18 in maintaining IGF-II B-chain helical stability was revealed in this study, with a lower helical content observed for all purified analogues. The lower helical content of Q18I IGF-II is consistent with the known destabilization of the helical conformation by the branched side chain of isoleucine^33^. Methionine and tyrosine are evidently not as favorable as glutamine at this position in IGF-II, perhaps due to their larger bulk compared to glutamine. Q18Y IGF-II was also noticeably less thermodynamically stable. Whilst tyrosine is present in the equivalent position in insulin there are differences in the i, i + 3 interactions, with a glutamate and asparagine in the i + 3 position of insulin and IGF-II, respectively. These differences may account for the tolerance of tyrosine at this position in insulin with respect to thermodynamic stability.

Molecular details of the specificity of IR and IGF-1R binding have been elucidated through the analysis of the three IGF-II Q18 analogues. Earlier mutagenesis of the IR residue F39 to serine (found at the equivalent IGF-1R residue S35)^34^ or alanine^34^ pointed towards a role of F39 in insulin binding. However, mutagenesis of the IGF-1R S35 to alanine did not disrupt IGF-I binding^35^. It was not until recent structures of the insulin:IR^21^ and IGF-II:IGF-1R^22^ complexes were solved that it became clear this may be a site of difference in the binding mechanisms at each receptor and a determinant of ligand receptor binding specificity. Site-directed mutagenesis of insulin pointed towards this^14,15,28^ but until now this has not conclusively been demonstrated using IGF-II.

Here we show that Q18 does not play a major role in IGF-1R binding as there was very little impact of the IGF-II Q18 substitutions on IGF-1R binding despite the biophysical changes observed. This is interesting given that structural analyses show Q18 is adjacent to side chains of L33, S35 and R59 IGF-1R^22^, but supports the S35A IGF-1R mutagenesis data showing no effect on IGF-I binding^35^. Consistent with this, the equivalent residue Q15 of IGF-I is in close proximity to the IGF-1R residues L33 and R59 in the IGF-I:IGF-1R structure^36^ and IGF-I mutation of Q15 to serine^37^, alanine^38^ or glutamate^38^ had little effect on IGF-1R binding (1.25, 2 and 1.4 fold decrease respectively). Thus, we have now conclusively demonstrated that these interactions are not contributing greatly to the overall IGF-1R site 1 binding affinity.

Structures of the insulin:IR-A reveal a π–π interaction between insulin YB16 and IR F39 (Fig. 1D)^21^. The importance of this π–π interaction for high affinity ligand binding to the IR was previously highlighted by mutation of insulin YB16 to glutamine or alanine which significantly reduced binding affinity^17,28^, whereas mutation to phenylalanine or tryptophan only lead to a modest change in IR affinity, presumably as the π–π interaction was maintained^39^. Here, through introduction of a tyrosine at residue 18 of IGF-II we show that by emulating the π–π interaction between insulin YB16 and IR F39 and we greatly increased the affinity of IGF-II for both IR-A and IR-B (Fig. 4B,C respectively). Indeed, the single residue change of Q18 to tyrosine was sufficient to confer to IGF-II the same IR-A binding affinity as insulin, whereas substitution with isoleucine, methionine (Fig. 4B) or alanine^18^ had little effect. Gauguin et al.^17^ reported that a sextuple IGF-II analogue (T7H, T16A, Q18Y, F48T, S50I, T58N^17^ IGF-II) had a similar affinity to insulin for the IR-A, and our current study would suggest that the Q18Y substitution is largely responsible for its increased binding affinity. The affinity of IGF-I for the IR was also increased by introduction of a tyrosine at the equivalent position in the Q15Y, F16L IGF-I analogue^40^, again highlighting the importance of a π–π interaction at this position. Overall, the evidence provided here conclusively demonstrates the key role of the interaction between insulin YB16 and IR F39 in high affinity binding and receptor binding specificity.

Following the initial binding event insulin and IGF-II activate the PI3K/Akt and ERK/MAP kinase signalling pathways with potencies in line with their IR-A binding affinities^41^. To our surprise, despite Q18Y IGF-II binding IR-A with equal affinity to insulin and an 8.2-fold higher affinity than IGF-II it was only able to activate Akt and ERK1/2 signaling to the same extent as IGF-II. This was also reflected in the DNA synthesis assay where Q18Y IGF-II was equipotent with IGF-II, Q18I IGF-II and Q18M IGF-II, with a ~ sevenfold lower potency compared to insulin (Fig. 6). We conclude that the formation of a π–π interaction with IR F39 essentially made no impact on the signalling outcome of IGF-II.

**Figure 7:**
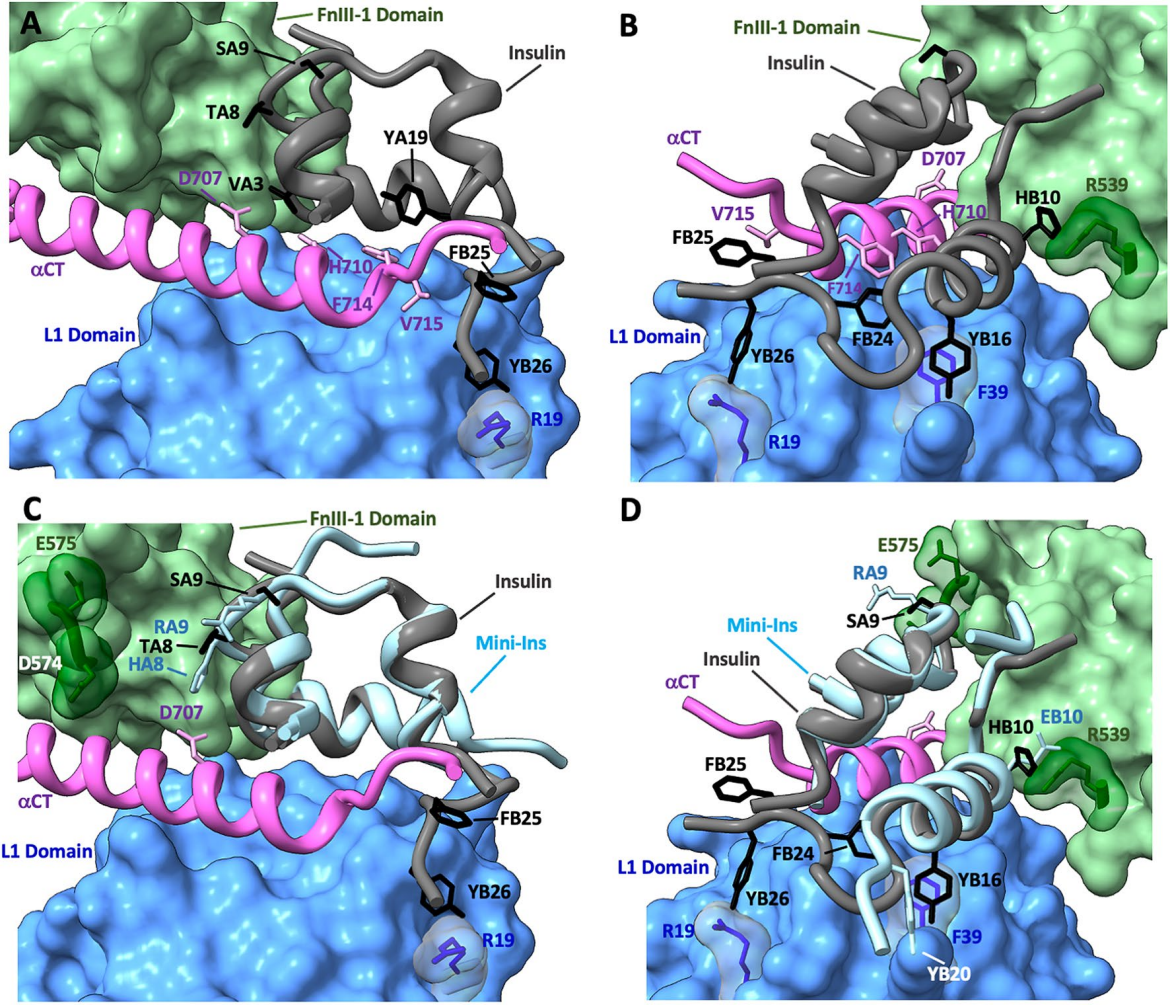
(**A**,**B**) CryoEM structure of insulin bound to the IR (PDB: 6HN5) highlighting the site 1 interaction. The surface filled L1 domain (cornflower blue) has residue R19 and F39 side chains shown (dark blue) and the αCT’ (purple) has residues H710, F714 and V715 side chains (light pink) depicted. The surface filled FnIII-1’ domain (light green) has R539 chain shown (dark green). Insulin binds to the IR primary binding site (αCT’ and L1 domain of opposite monomers), with key contacts involving insulin residues VA3, YA19 and FB25 with αCT’ resides H710, F714 and V715. Additionally, insulin residues FB24, FB25 and YB26 (FFY motif) contact residues R19 and YB16 of the L1 domain. An additional interaction is made between insulin residue HB10 and R539 of the FnIII-1’ domain. (**C**,**D**) CryoEM structure of insulin bound to the IR with mini-ins overlayed (PDB: 6HN5 and 6VET respectively). The lack of the FFY motif in mini-ins is compensated through GB20Y mutation which likely interacts with residue F39 of the L1 domain. The interaction with FnIII-1’ domain R539 is strengthened through a HB10E mutation and additional contacts are made through residue E575 and mini-ins residues SA9R and TA8H.

We have sought to explain why Q18Y IGF-II does not activate the IR proportionately to its binding affinity through the knowledge provided by recent cryoEM structures of insulin bound to the IR, as there is currently no structure available of the IGF-II:IR complex. In what is believed to be the fully activated IR structure, insulin binds at site 1 to the IR L1 domain from one monomer and the αCT’ and FnIII-1’ domains from the opposite monomer^21^. Of the contacts made between insulin residues and IR site 1, most involve side chains of residues in either the αCT’ alone, both the αCT’ and L1 or the FnIII-1’ (Fig. 7A,B). It is uncommon for contact to be made only with the L1 domain, as is the case for the tyrosine π–π interaction with IR F39. Mutation of insulin residues primarily binding the αCT’ negatively affect αCT’ interaction and significantly reduce IR activation^42^. This is seen, for example, upon mutation to alanine of A-chain insulin residues VA3 and YA19 that contact IR residues F710 and F714, and residue FB25 that contacts IR V715. Similarly, simultaneous binding of insulin to both IR monomers through αCT’ and L1 interaction is critical for high affinity binding and IR activation^43^. Insulin FB24 within the critical FB24, FB25, YB26 motif is regarded as a critical anchor residue that contacts αCT’ F714 and the L1 domain^44^.

Further interactions between insulin residues and the FnIII-1’ contribute to the overall binding affinity and can influence activation and downstream signalling. For example, HB10 approaches IR FnIII-1’ residue R539 in the fully activated structure, and mutation of HB10 to asparagine^45^ or glutamate^46–48^ results in two–fourfold increased IR affinity leading to greater metabolic potency. Similarly, the recently developed mini-ins analogue (HA8, RA9, EB10, YB20 des-octapeptide) designed based on structural features of a cone snail insulin-like peptide^49,50^ has increased FnIII-1’ affinity that results in altered signalling. Not only does mini-ins residue EB10 form the salt bridge with R539 (Fig. 7D)^49^ but residues HA8 and RA9 make additional contacts with FnIII-1’ domain residues D574 and E575, respectively, that are not observed in the insulin:IR complex (Fig. 7C). The strengthened affinity for the FnIII-1’ domain results in equal IR binding affinity to insulin by compensating for the lack of the site 1 B24 anchor residue. Mini-ins has a potency in Akt activation similar to insulin but a reduced ability to activate ERK1/2.

Unlike FnIII-1’ interactions, evidently in the case of Q18Y IGF-II increasing the affinity through the L1 domain interaction alone does not influence IR signalling. However, by introducing the Q18Y change and concomitantly strengthening the interaction at the FnIII-1’, as is seen with the sextuple IGF-II analogue, signalling potency can be increased to equal that of insulin^17^. Other residues that contact the L1 alone, including SB9, EB21 and YB26 (YB26 shown in Fig. 7), also don’t contribute greatly to IR binding affinity or biological activity^46,51,52^, supporting the proposal that an L1 only interaction has a lesser impact on activation than the interactions with the αCT’ and FnIII-1’ domains of the opposite monomer. In conclusion, our findings provide some insight into the mechanism of IR activation enabled through specific ligand:receptor interactions. However, a full understanding of the mechanisms driving signalling bias will require further investigation.

## Experimental procedures

### Cell lines and culture conditions

hIR-A and hIR-B overexpressing cells (R^-^IR-A and R^-^IR-B, respectively) were constructed as described^31^ using R-fibroblasts (derived from IGF-1R knockout mouse embryonic fibroblasts), a kind gift from Professor R. Baserga (Philadelphia, PA)^53^. L6 IR-A cells were kindly provided by Dr. B. F. Hansen (Novo Nordisk A/S, Denmark)^54^. P6 cells (BALB/c3T3 cells overexpressing the human IGF-1R) were from Professor R. Baserga^55^. All cells were maintained at 37 °C in 5% CO2. R-IR-A, R-IR-B, L6 IR-A, and P6 cells were maintained in Dulbecco’s modified Eagle’s medium high-glucose (4.5 g/ml, DMEM) supplemented with 10% fetal calf serum, 100 units/mL penicillin, 100 μg/mL streptomycin and 0.5% (250 μg/mL) G418. All cell culture media and supplements were purchased from Thermo Fisher Scientific Australia. Fetal calf serum is from Bovogen.

### Construction of Expression Plasmids Encoding Human IGF-II Analogues

The cDNA encoding IGF-II and analogues was digested with XbaI and EcoRI restriction enzymes and ligated into the pET32a expression vector, in frame^56^. Each construct was transformed into Escherichia coli BL21 cells for expression. IGF-II peptide expression was induced by isopropyl β-D-thiogalactoside. IGF-II peptides were expressed with a fusion partner comprising the first 11 amino acids of porcine growth hormone ([Met1]pGH-(1–11)) as previously described^57^.

### Refolding and Purification of IGF-II Analogues

As described^58^, washed pGH (1–11) IGF-II inclusion bodies (harvested from either a 0.5 or 1 L ferment) were solubilized in 8 M urea, 40 mM glycine, 0.1 M Tris, and 20 mM dithiothreitol (DTT) at pH 2.0. The solubilised inclusion bodies were separated on a Superdex 75 size exclusion chromatography column (10 × 300 mm (GE Healthcare) using the same buffer containing 1.6 mM DTT. Fractions were screened for pGH (1–11) IGF-II protein using analytical C4 HPLC and SDS-PAGE. Fractions containing pGH (1–11) IGF-II protein were pooled and diluted to 0.1 mg/mL in 2.5 M urea, 12.5 mM glycine, 0.7 M Tris, 5 mM EDTA, 0.5 mM dithiothreitol, 1 mM 2-hydroxyethyl disulfide, pH 9.1. Refold was monitored by analytical C4 reverse phase HPLC. The pGH (1–11) fusion partner was cleaved with α-lytic protease and then separated from each IGF-II analogue by reverse-phase HPLC as previously described^37,59^. Final purified proteins were analyzed by mass spectroscopy.

### Circular dichroism

Circular dichroism (CD) was performed as previously described^60^. Briefly, CD spectra were recorded on a Jasco J-1500 CD spectrometer. Spectra were from 300 to 180 nm with a 1.0 nm step size using a 1.0 s response time and 1.0 nm bandwidth in a quartz cuvette with a 0.2 cm path length. IGF-II and IGF-II analogues were resuspended in 10 mM acetic acid (pH 3) to a concentration of 0.05 mg/mL. To correct for background, the spectrum of buffer alone was subtracted from each sample spectra. Temperature denaturation studies were conducted by automated thermal control increasing by 2 °C/min at 1 °C intervals at a peptide concentration of 0.05 mg/mL in 10 mM acetic acid (pH 3). The machine units collected, θ in millidegrees, was converted to the mean residue ellipticity (MRE), [θ] in degrees.cm^2^dmol^−1^residue^−1^, as follows:

$$\left[\theta \right]=\theta \times \frac{(0.1 \times MRW)}{(P \times Conc.)}$$

The MRW is the protein mean weight ((atomic mass units/daltons)/number of residues)), P is pathlength (cm) and Conc. is protein concentration in mg/mL. [θ]_222_ is the molar ellipticity per residue at wavelength 222 nm. Helical content was calculated using the CDSSTR algorithm^61^ for deconvolution against the reference protein database set SMP180. The program is available on the DICROWEB website (http://dichroweb.cryst.bbk.ac.uk/html/home.shtml).

### IGF-1R and IR-binding assays

IGF-1R, IR-A and IR-B binding was measured essentially as described^31^. Human IGF-1R, IR-A or IR-B were solubilized from P6, R-IR-A and R-IR-B cell lines, respectively. Cells were serum-starved in serum-free medium (SFM) containing 1% BSA for 4 h prior to lysis in ice–cold lysis buffer (20 mM HEPES, 150 mM NaCl, 1.5 mM MgCl_2_, 10% (v/v) glycerol, 1% (v/v) Triton X-100, 1 mM EGTA, cOmplete protease inhibitor cocktail (Roche), pH 7.5) for 1 h at 4 °C. Cell lysates were centrifuged at 2200*g* for 10 min, then 100 μl of lysate was added to each well of a white Greiner Lumitrac 600 96-well plate, pre-coated with anti-IGF-1R antibody 24–31 or anti-IR antibody 83–7 (250 ng/well in bicarbonate buffer, pH 9.2).

Approximately 1,500,000 fluorescent counts of europium-labelled IGF-II or insulin (Eu-IGF-II or Eu-Insulin) were added to each well along with increasing concentrations of unlabelled competitor in a final volume of 100 μl and incubated for 16 h at 4 °C. Wells were washed three times with 20 mM Tris, pH 7.4, 150 mM NaCl, and 0.1% (v/v) Tween 20 (TBST). Finally, 100 μl/well DELFIA enhancement solution (PerkinElmer Life Sciences) was added to each well for 10 min before measuring time-resolved fluorescence (measured with 340-nm excitation and 612 nm emission filters by a Victor X4, 2030 Multilabel Reader (Perkin Elmer). Two (IGF-IR binding), four to six (IR-A binding) and three (IR-B binding) independent assays were performed, each comprising three technical replicates per data point. Mean IC_50_ values were calculated with the statistical software package Prism v9.0.0 (GraphPad Software) after curve fitting with a nonlinear regression (one-site) model.

### Signalling activation

Following a starvation period of 4 h in serum free DMEM, L6 IR-A cells were stimulated with 10 nM IGF-II or Q18 IGF-II analogues (Q18I, Q18M, Q18Y) for various times (up to 20 min in DMEM containing 1% bovine serum albumin). L6 hIR-A cells express 287,000 IR-A receptors compared to 25,800 IGF-1R receptors, meaning the IR-A homodimer is the predominant receptor responding to IGF-II and analogues in this cell line^62^. Lysates of cells were trichloroacetic acid precipitated and subjected to separation on reducing 10% SDS-PAGE gels, transferred to nitrocellulose membranes and immunoblotted with primary antibodies for 16 h. Antibodies used were phospho Akt (T308) (New England Biolabs #9275S), phospho p44/42 MAPK (ERK1/2) (T202/Y204) (New England Biolabs #9101S) and mouse anti-β-tubulin (Invitrogen #32–2600), IRDye 800CW donkey anti rabbit IgG (Millenium Science 926-32213), IRDye 680RD donkey anti mouse (Millenium Science 926-68072). Total Akt and ERK1/2 levels do not change over the time course measured (data not shown). Blots were scanned using an Odyssey CLx Imaging System (LI-COR Biosciences). Quantitation was performed with Image Studio Lite software. β-tubulin was used as a loading control and data was normalised to this. Activation is expressed as a fraction of the response to IGF-II at 20 min. A total of four independent experiments were performed and the data is an average of these.

### DNA synthesis assays

DNA synthesis was carried out based on the labelling approach previously described with some modifications^63^. L6 IR-A cells were plated in a 96 well flat bottom plate (32 × 104 cells/well) and grown overnight at 37 °C, 5% CO_2_ as previously described^17^. Briefly, cells were starved in SFM for 2 h prior to treatment with increasing concentrations of insulin, IGF-II or IGF-II analogues (0.01—300 nM) for 18 h in DMEM, 1% BSA at 37 °C and 5% CO_2_. The cells were incubated with 10 µM of 5-Ethynyl-2’-deoxyuridine (EdU) for 4 h, washed with filtered PBS, 1% BSA and fixed in the dark for 15 min with 4% paraformaldehyde (PFA). Fixed cells were washed with PBS, 1% BSA and permeabilised for 20 min with 0.5% Triton X-100. A click chemistry labelling cocktail (2 µM FAM-Azide 488, 100 mM Tris, pH 7.5, 4 mM CuSO_4_, 100 mM sodium ascorbate) was added to the cells for 30 min at room temperature in the dark. Finally, cells were washed thrice with PBS, 1% BSA and fluorescence was measured using 485 nm excitation and 535 nm emission filters with a PerkinElmer VICTOR X4 2030 Multilabel Reader. Assays were performed in triplicate in at least three independent experiments.

### Statistical analysis

Statistical analyses of receptor binding, receptor activation, and DNA synthesis assays were performed using a two-way ANOVA with Dunnett’s multiple comparison. Significance was accepted at p < 0.05.

## Supplementary Information

Supplementary Information.
