# Supplementary material for: Determinants of IGF-II influencing stability, receptor binding and activation
Source: Sci Rep. 2022 Mar 18;12:4695. doi: 10.1038/s41598-022-08467-8 (PMC8933565; doi:10.1038/s41598-022-08467-8)
Supplement: Supplementary file 1 — Supplementary Information. [file 41598_2022_8467_MOESM1_ESM.docx]

Supplementary Information

**Determinants of IGF-II influencing stability, receptor binding and activation**

**Andrew Blyth^1^, Michael Ortiz^1^, Allanah Merriman^1^, Carlie Delaine^1^, and Briony Forbes^1^,^*^**

^1^Department of Medical Biochemistry, Flinders Health and Medical Research Institute, Flinders University, Bedford Park 5042, South Australia, Australia

****^*^Correspondence to briony.forbes@flinders.edu.au

**Figure S1.** Coomassie-stained reducing 15% tricine SDS-PAGE of Q18I IGF-II, Q18M IGF-II and Q18Y IGF-II protein expression in E. coli BL21 cells. Non induced (-) and Isopropyl β-d-1-thiogalactopyranoside (IPTG) induced (+) whole cell lysates.

**Figure S2.** (A) Analytical HPLC profiles of IGF-II from dissolution to final folded form. (A) Dissolution sample of pGH (1-11) IGF-II peptide. (B) Gel filtration pool of pGH (1-11) IGF-II peptide. (C) Refold t=0 (black), t=20 hours (red) samples of pGH (1-11) IGF-II and t=31 hour post cleavage of IGF-II. (D) Final purified IGF-II.

**Figure S3.** (A) Analytical HPLC profiles of Q18I IGF-II from dissolution to final folded form. (A) Dissolution sample of pGH (1-11) Q18I IGF-II peptide. (B) Gel filtration pool of pGH (1-11) Q18I IGF-II peptide. (C) Refold t=0 (black), t=20-hours (red) samples of pGH (1-11) Q18I IGF-II and t=12-hours post cleavage of Q18I IGF-II. (D) Final purified Q18I IGF-II.

**Figure S4.** (A) Analytical HPLC profiles of Q18M IGF-II from dissolution to final folded form. (A) Dissolution sample of pGH (1-11) Q18M IGF-II peptide. (B) Gel filtration pool of pGH (1-11) Q18M IGF-II peptide. (C) Refold t=0 (black), t=20 hours (red) samples of pGH (1-11) Q18M IGF-II and t=15 hours post cleavage of Q18M IGF-II. (D) Final purified Q18M IGF-II.

**Figure S5.** Analytical HPLC profiles of Q18Y IGF-II from dissolution to final folded form. (A) Dissolution sample of pGH (1-11) Q18Y IGF-II peptide. (B) Gel filtration pool of pGH (1-11) Q18Y IGF-II peptide. (C) Refold t=0 (black), t=20 hours (red) samples of pGH (1-11) Q18Y IGF-II and t=12 hours post cleavage of Q18Y IGF-II. (D) Final purified Q18Y IGF-II.

**Figure S6.** Uncropped Akt/ ERK activation Western Blot shown in Fig. 5A and 5B.

**Table S1.** Matrix assisted laser desorption and ionization mass spectrometry (MALDI MS) of IGF-II, Q18I IGF-II, Q18M IGF-II, Q18Y IGF-II.
